# Colostrum traits and newborn body weight and growth: comparison between single and twin underfed sheep pregnancies

**DOI:** 10.3389/fvets.2023.1256989

**Published:** 2023-09-08

**Authors:** Jesús Turín, Francisco Sales, Oscar A. Peralta, Mónica De los Reyes, Consuelo Borie, Albert Carrasco, Antonio González-Bulnes, Víctor H. Parraguez

**Affiliations:** ^1^Magister in Animal and Veterinary Sciences Program, Faculty of Veterinary Sciences, University of Chile, Santiago, Chile; ^2^CRI-Kampenaike, INIA, Punta Arenas, Chile; ^3^Faculty of Veterinary Sciences, University of Chile, Santiago, Chile; ^4^Facultad de Ciencias Veterinarias, Universidad de Concepción, Chillán, Chile; ^5^Facultad de Veterinaria, Universidad CEU Cardenal Herrera, CEU Universities, Valencia, Spain; ^6^Faculty of Agrarian Sciences, University of Chile, Santiago, Chile

**Keywords:** pregnancy outcome, extreme environment, undernourishment, immunoglobulin G, newborn survival

## Abstract

Maternal nutrition during gestation plays an important role in colostrum production, postnatal growth, and survival of newborn lambs, especially in twin gestations. This research aimed to investigate the effects of chronic natural undernutrition on colostrum traits and early lamb’s postnatal growth born from single and twin sheep pregnancies developed in a restrictive prairie, representative of southern Patagonia. Single- and twin-bearing ewes (*n* = 20 per group) were maintained grazing in a natural pasture. At 140 days of gestation, ewes were placed in individual pens for lambing control. Colostrum was collected immediately after delivery and at 12, 24, and 36 h postpartum, for determination of yield and composition. Maternal blood was obtained at 140 days of gestation and at lambing for plasma glucose, progesterone, 17β-estradiol, and IgG determination. Newborn lamb blood for determining glycaemia and IgG was collected at birth and at 12, 24, 36, and 120 h after birth. Lamb mortality and growth was assessed from birth until 30 days of life. No differences were observed in progesterone and 17β-estradiol. There were no differences in colostrum yields and fat components, however single- had higher values of protein and lactose than twin-bearing ewes (*p < 0.05* for both). Singletons had higher glycaemia than twins at 12 h postpartum (102.2 ± 32.8 vs. 73.4 ± 29.9 mg/dL, *p < 0.05*). Colostrum IgG content was similar at delivery but higher in single ewes at 12 and 24 h, reaching a similar values at 36 h (4.7 ± 9.7 and 5.8 ± 7.7 mg/mL in single and twin pregnancies, respectively). Newborn IgG was higher in singletons compared to twins at least until 48 h of life. Lams body weight was always superior in singleton than twins from birth until 30 days of life. Mortality did not differ during the first week of life, but it increased significantly only in twins until day 30 of life. Undernourishment in pregnant ewes affected colostrum quantity and quality, resulting in a lower postnatal growth and a higher mortality in twins. Alternative managements favoring fetal growth, birth weight and neonatal viability in twin sheep pregnancies are needed, when flocks are breed under harsh environments.

## 1. Introduction

Sheep is a key global economic resource because of its great adaptability to different climatic, altitudinal and nutritional conditions, greater than any other animal species. Sheep breeding is mainly carried by smallholders from developing and transition regions, taking place in marginal territories with extreme conditions and little availability of water and pasture (1). As an example, a traditional area for sheep breeding in South America is the Magellan Patagonia (2, 3) which is a semi-arid region where climatic conditions are extreme, with low temperatures, strong winds, and low rainfall.

Extensive sheep systems found in arid or semi-arid lands, such as those encountered in Patagonia, offer insufficient nutrient availability to cover animal demands. In these scenarios, the main limiting factor for sheep breeding is maternal undernutrition during pregnancies (4). In turns, maternal undernutrition may affect viability and development of the newborn lamb because results in restricted pregnancies with low birth-weight (5, 6) and high perinatal mortality of the lambs, mainly in twin pregnancies (7). Most of the perinatal deaths occur mainly during the first 3 days after birth, because of starvation, as a result of maternal undernutrition and poor resistance to inclement weather (8) but also because undernourished ewes have a lower quantity and quality of colostrum, reducing the chances of postpartum lamb’s survival (9); even more in case of increased prolificacy, with twin pregnancies.

In spite of the fact that, currently, sheep breeders aim to increase efficiency by increasing prolificacy and the fact that sheep are still bred extensively in territories with little nutritional supply, the effect of twinning and undernutrition on colostrum yield and components has been scarcely studied. Previous studies in twin pregnant ewes have shown that colostrum yield and its total content of solids are higher than in single pregnancies, but with very little changes in other components (protein, fat, and lactose) (10). In single ovine pregnancies, maternal undernutrition during the last two thirds of gestation (covering only 60% of total requirements for pregnancy) decreases total colostrum yield and density but increases IgG content, without significant variations in contents of protein, fat, lactose and non-fat solids (11). There is only an experimental approach which assessed combined effects of nutrition and twinning (12), by supplying twin pregnant ewes with a diet that reduced its glycaemia by about 50% from 112 days of gestation; data indicated that colostrum yield during the first 18 h after lambing was reduced by ~50%, but its nutritional components did not undergo changes.

Hence, in view of these previous considerations, the aim of the present study was to assess the effects of chronic natural undernutrition on colostrum traits and early postnatal growth of the lambs born from single and twin pregnancies in sheep kept grazing on a restrictive prairie, representative of southern Patagonia.

## 2. Materials and methods

### 2.1. Ethics statement

The sheep general handling and the experimental protocols developed in this work were approved by the Bioethics Committee of the Faculty of Veterinary, University of Chile, and the Institutional Committee for the Care and Use of Animals (Certification # CICUA 20394-VET-UCH).

### 2.2. Location, animals, and management

The study was carried out at the Kampenaike Regional Research Center, belonging to the Agricultural Research Institute (INIA), Ministry of Agriculture, located 60 km from Punta Arenas city, in southern Patagonia (Magallanes and Chilean Antarctic Region, latitude 52° 36′; longitude 70° 56′).

A total of 70 Corriedale ewes (4–6 years old) of similar weight and body condition were initially selected from the INIA commercial flock. Ewes were subjected to estrous synchronization and superovulation by the use of an intravaginal device containing 0.3 g of progesterone (CIDR G^®^, Pfizer, Chile) for 11 days plus the administration of an intramuscular dose of a prostaglandin F_2α_ (PGF_2α_) analog (125 μg cloprostenol, Estrumate^®^, ICI, Macclesfield, United Kingdom) at CIDR insertion. Afterward, an i.m. dose of 400 IU of equine chorionic gonadotropin (eCG, Novormon^®^, Syntex, Argentina) was applied at CIDR removal. These females were maintained during 5 days with three fertility-proven Corriedale rams for allowing natural mating. The rams had their chests impregnated with paint to mark the mated sheep and to recognize the day of mating.

Afterward, at 60 and 90 days after mating, assessments of body weight (BW) and body condition score (BCS), using the 0–5 scale (13), was subsequently performed in all the sheep. At Day 90 after mating, type of pregnancy (single or twin) was determined in all the animals by ultrasonography and 40 sheep carrying single (*n* = 20) and twin pregnancies (*n* = 20) were selected. These ewes were kept grazing on natural pasture representative of Patagonian prairies (*Festuca gracillima*-*Chiliotrichium diffusum*; Crude Protein: 3.3%, Metabolizable Energy: 1.9 Mcal/kg, Total Digestible Nutrients: 45%, dry matter availability of 525 kg per hectare), in a paddock with a stocking rate of 0.9 ewes per hectare. Assessments of BW and BCS was performed again at 120 and 140 days of pregnancy.

### 2.3. Maternal and newborn sampling

The ewes were placed in individual pens on day 140 of gestation, for surveillance of parturitions and samplings, and fed with 1.6 and 2.0 kg of alfalfa hay of per day for single and twin pregnancies, respectively; water was available *ad libitum*. On that day and immediately after lambing (within a period of about 1 h), maternal blood samples (5 mL) were obtained from the jugular vein to measure glycemia, progesterone, estradiol-17β, and IgG.

Samples of colostrum were obtained, immediately after delivery and in any case before the lambs started lactation, by complete manual milking of one of the mammary glands, after i.m administration of oxytocin (5 IU; Veterinary Pharmacology FAV, Chile). Total amount of colostrum was determined by collecting it in a graduated container and two colostrum samples were separated; one sample of 1.5 mL was frozen in liquid nitrogen for the IgG assessment and one sample of 40 mL was mixed with 100 μL of 10% potassium dichromate in Falcon tubes and used for nutritional composition analysis. The remaining colostrum was fed to the lambs in bottles and the contralateral teat was allowed to be suckled by lambs. After that, such contralateral teat was covered with adhesive tape in order to prevent suckling by lambs and used to measure colostrum production at the next milking. The colostrum sampling was performed at 12, 24, and 36 h postpartum, by alternatively milking both teats. Total colostrum yield at each time was estimated by multiplying by two the volume obtained from the milked gland.

At birth, the lambs were dried, sexed and weighed (Camry^®^ digital scale, China), and a venous blood sample (5 mL) was taken. Glycaemia and IgG were measured immediately and the remaining blood sample was centrifuged (1,300 × *g* for 5 min) and the plasma obtained was frozen in liquid nitrogen for later analysis of cortisol. Venous blood samples for determining glycaemia and IgG were obtained also at 12, 24, 36, and 120 h after birth. The body weight and mortality of the lambs was evaluated at birth and on days 7 and 30 after birth.

### 2.4. Samples analysis

In both maternal and newborn blood, glycaemia was determined immediately after sample collection, by using a portable enzymatic colorimetric method (OneTouch^®^ glucometer, LifeScan, Inc., Switzerland). This method has been previously validated for its use in sheep (14).

The IgG concentration was measured both in colostrum and in lamb’s blood plasma samples by radial immunodiffusion (RID), in accordance to the technique proposed by Fahey and McKelvey (15) and Mancini et al. (16), and modified by Waldner and Rosengren (17). Briefly, 3 g of sheep IgG-specific antibodies (rabbit Anti-Sheep IgG, whole molecule, S1265, Sigma-Aldrich Co, St. Louis, United States) were incorporated into 100 mL of 1% agarose gel, pH 7.4, heated to 56°C and, then, 15 mL of the solution were poured into petri dishes for cooling. After cooling and gelling of the agarose, 2.5 mm diameter wells were made, equidistant and 3 cm apart. After cooling and gelation of the agarose, 2.5 mm diameter wells were made Afterward, 4 μL of blood or colostrum samples (previously diluted 1:10 and 1:15 for plasma and colostrum, respectively) were added into the wells. After incubation for 24 h at 20°C in a humid chamber, the diameters (mm^2^) of the diffusion halos were measured. Previously, using the same procedure, a calibration curve was made by using known concentrations of sheep IgG (IgG from sheep serum, I5131, Sigma-Aldrich Co, St. Louis, United States; 1.25; 2.5; 5.0; 7.0; and 10.0 mg/mL) and performing a linear regression equation between the IgG concentration and the squared diameter of the precipitate halos. Hence, such equation was used to determine the concentration of IgG in colostrum and plasma samples.

Progesterone, 17β-estradiol, and cortisol in blood plasma samples were measured by solid phase radioimmunoassays. Briefly, progesterone was determined in 50 μL plasma aliquots, using the PROG-RIA-CT (KIP1458, DiaSource ImmunoAssays, Louvain-la-Neuve, Belgica). The quantification limit for the assay was 0.3 ng/mL and the coefficients of variation intra-assay was 0.67–4.48%. Estradiol-17β was assessed in 100 μL plasma aliquots, using the E_2_-RIA-CT (KIP0629, DiaSource ImmunoAssays, Louvain-la-Neuve, Belgica). The quantification limit for the assay was 3.44 ng/mL and the coefficients of variation intra-assay was 0.22–3.96%. Cortisol was measured in 25 μL plasma aliquots, using the Cortisol-RIA-CT (KIPI28000, DiaSource ImmunoAssays, Louvain-la-Neuve, Belgica). The quantification limit for the assay was 8.57 μg/L and the coefficients of variation intra-assay was 0.03–2.22%.

Finally, colostrum composition (fat, protein, and lactose) was evaluated in an Ekomilk^®^ analyzer (Milkana KAM98-2A, Bulteh 2000 Ltd., Stara Zagora, Bulgaria). Samples were heated to 38°C and homogenized on a shaker for 3 min. Then, they were diluted in deionized distilled water (1:1 v/v), quickly homogenized and evaluated at 23°C.

### 2.5. Statistical analysis

The sample size was statistically calculated according to the variability of colostrum production at lambing (18), considering a statistical power of 95% and *α* = 0.05. The variables measured consecutively in time were compared by ANOVA, using a general linear model for repeated measures (GLM; SAS Institute Inc., Cary, NC, United States), considering time as a fixed effect, after performing the data normality test. The Bonferroni comparison was used as a *post-hoc* test to determine the presence of significant differences between groups. In the case of data that did not follow the normal distribution, the Wilcoxon signed rank test was used for means comparison. For the comparison of means between single and twin pregnancies, the parametric Student’s T test or the non-parametric Mann–Whitney U test were used, according the data distribution. Comparison of mortality of the lambs between groups was done by means chi square analysis. Data are presented as means ± SDs. Significant differences were considered when *p ≤ 0.05*, and a tendency when *p > 0.05* and *≤ 0.1*.

## 3. Results

### 3.1. Ewes BW and BCS

**Figure 1:**
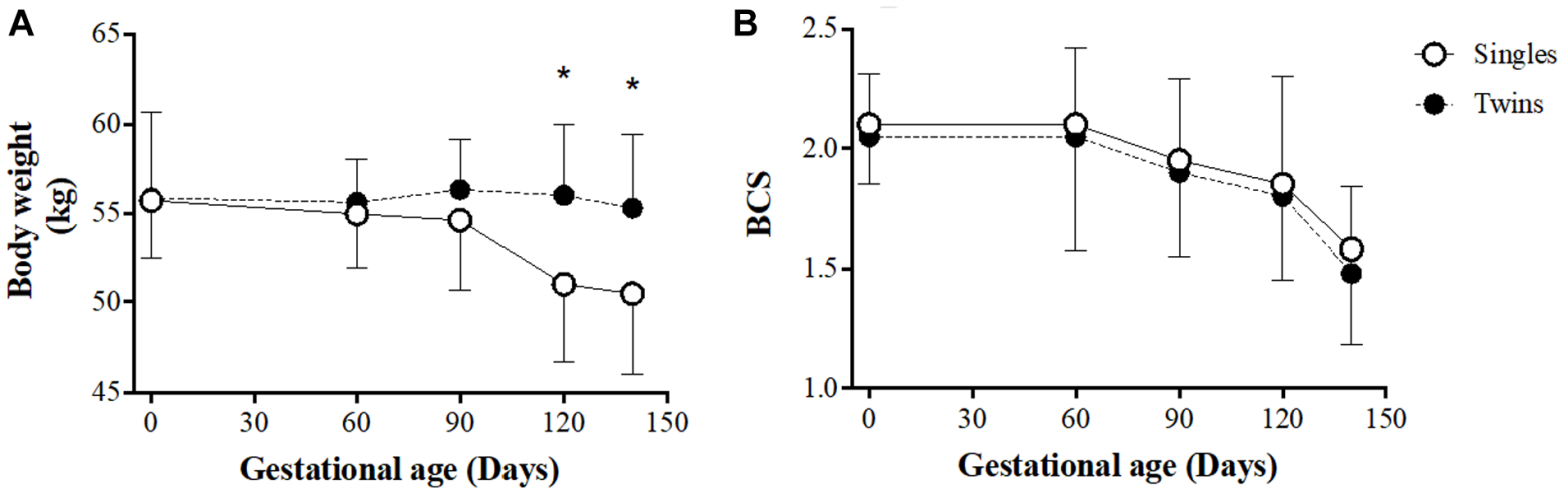
Body weight **(A)** and condition score **(B)** in single- and twin-bearing ewes in a model of pregnancy under natural undernutrition. Asterisks indicate significant difference (*p ≤ 0.05*) between groups at the same gestational age.

The evolution of BW and BCS of the ewes during gestation are shown in Figure 1, panel A and B, respectively. At the beginning of pregnancy, BW and BCSs were similar between groups (55.7 ± 3.2 kg and 2.10 ± 0.21; 55.9 ± 4.8 kg and 2.05 ± 0.28 for single and twin pregnancies, respectively; *p > 0.05*). BW in twin-bearing ewes remained almost steady throughout pregnancy while gradually decreased in single-bearing ewes, so differences were statistically significant from 120 days of gestation onwards (*p < 0.05*) and reached maximum difference at 140 days of gestation (50.5 ± 4.5 and 55.3 ± 4.6 kg BW for ewes carrying single and twin fetuses, respectively; *p < 0.05*). On the other hand, the score and evolution of BCS did not show differences between groups throughout pregnancy, remaining mostly stable between 0 and 60 days of gestation and decreasing therefore during the remaining time of pregnancy (1.58 ± 0.50 for single- and 1.48 ± 0.40 for twin-bearing ewes at day 140 of gestation; *p > 0.05*).

### 3.2. Maternal glycaemia and reproductive hormones

The concentrations of glucose, progesterone, and 17β-estradiol in maternal blood plasma at 140 days of gestation and immediately after lambing are shown in Table 1. Glycaemia and estradiol-17β concentrations sharply increased after parturition but no differences were observed between single- and twin-ewe pregnancies at any of the two evaluation times; however, prior to lambing, the glycaemia in twin-bearing ewes showed a trend (*p* = 0.092) to be lower than those carrying singletons. In contrast, plasma progesterone concentrations showed a pronounced decrease after lambing and a trend for being higher in twin pregnancies at 140 days of gestation (*p = 0.061*), but without significant differences at postpartum.

**Table 1:** Concentrations of glucose, progesterone, and estradiol-17β in blood plasma samples obtained at day 140 of gestation (BP) and immediately after parturition (AP), in single- and twin-bearing ewes in a model of natural undernutrition.

	Glucose (mg/dL)		Progesterone (ng/mL)		17β-Estradiol (pg/mL)	
	BP	AP	BP	AP	BP	AP
Singles	41.6 ± 19.6	149.8 ± 41.5	62.1 ± 14.7	2.1 ± 0.8	10.6 ± 3.8	62.1 ± 40.8
Twins	29.4 ± 13.8	144.6 ± 37.5	75.8 ± 26.5	2.7 ± 1.3	12.0 ± 2.9	93.0 ± 48.0
*p value*	*0.092*	*ns*	*0.061*	*ns*	*ns*	*ns*

ns, not significant.

### 3.3. Colostrum yield and nutritional composition

Table 2 shows the accumulated colostrum yield and the average content of each nutritional component (protein, fat and lactose) during the first 36 h after lambing. There were no differences in yields and fat components between groups but sheep with single pregnancies showed higher values of protein and lactose than sheep carrying twin pregnancies (*p < 0.05* and *p = 0.05*, respectively).

**Table 2:** Colostrum yield during the first 36 h after lambing and colostrum composition as average of the sampled period (0–36 h after lambing) in single- and twin-bearing ewes, in a model of natural undernutrition.

	Accumulated colostrum yield (mL)	Protein (%)	Lactose (%)	Fat (%)
Singles	1245.1 ± 420.3	7.48 ± 3.75	5.61 ± 0.63	7.77 ± 3.39
Twins	1079.0 ± 403.4	6.15 ± 2.50	5.39 ± 0.49	7.80 ± 3.26
*p value*	*ns*	*0.046*	*0.053*	*ns*

ns, not significant.

**Figure 2:**
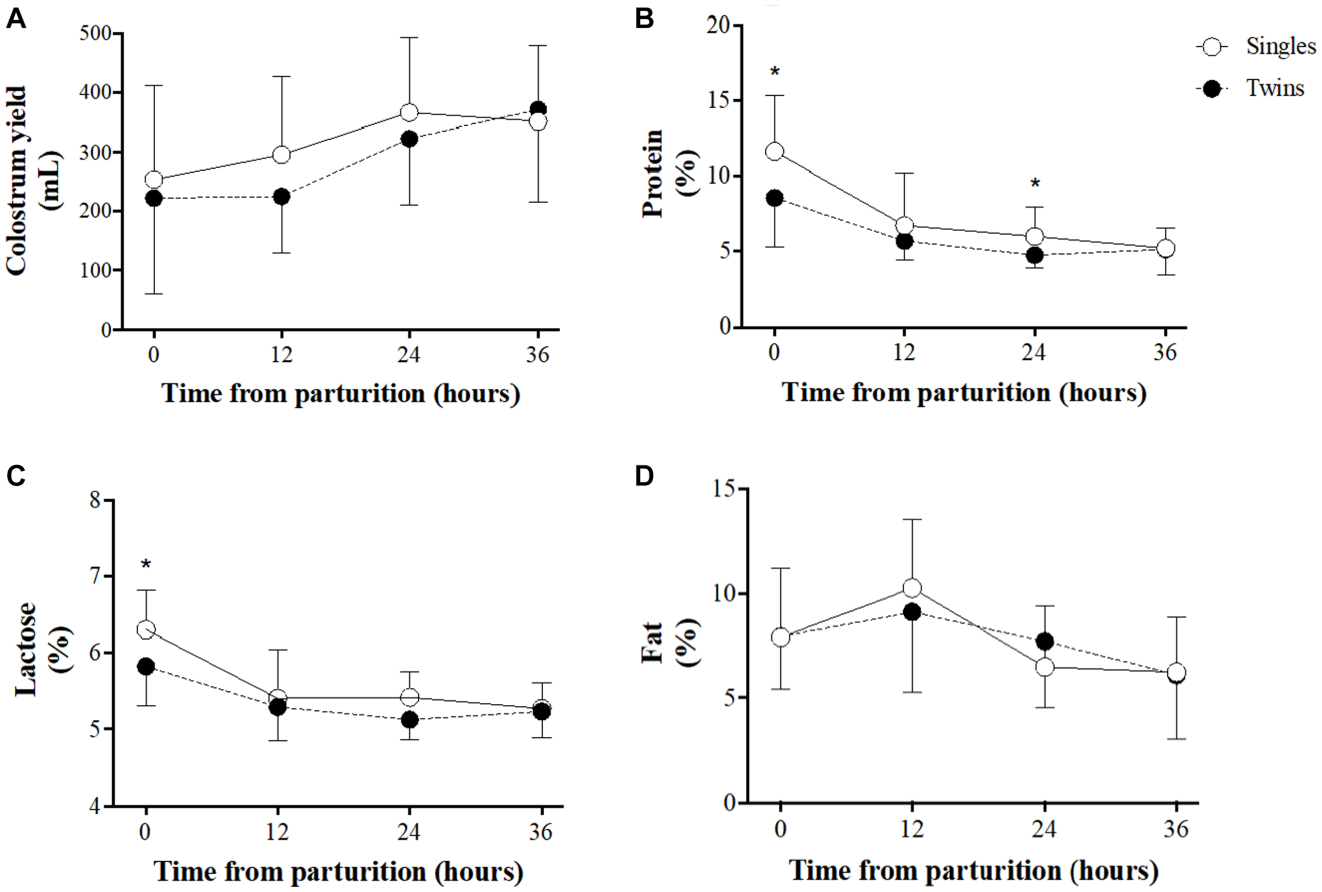
Colostrum traits in single- and twin-bearing ewes in a model of pregnancy under natural undernutrition. Colostrum yield **(A)**, % protein **(B)**, % lactose **(C)**, and % fat **(D)**. Asterisks indicate significant difference (*p ≤ 0.05*) between groups at the same sampling time.

A comparison of colostrum yields and composition between single- and twin-lambing ewes over time is shown in Figure 2. Colostrum yields (Figure 2A) increased during the first 36 h of lactation in both groups, without significant differences between groups over time. Assessment of colostrum components showed that protein and lactose content decreases over time in both groups (Figures 2B,C, respectively), showing higher values in single-lambing ewes at 0 h for both components and also at 24 h in the case of protein (*p < 0.05* for both groups). Fat content (Figure 2D) remained almost steady, with a slight increase during the first 12 h, without significant differences between groups.

### 3.4. Newborns blood glucose and cortisol

**Figure 3:**
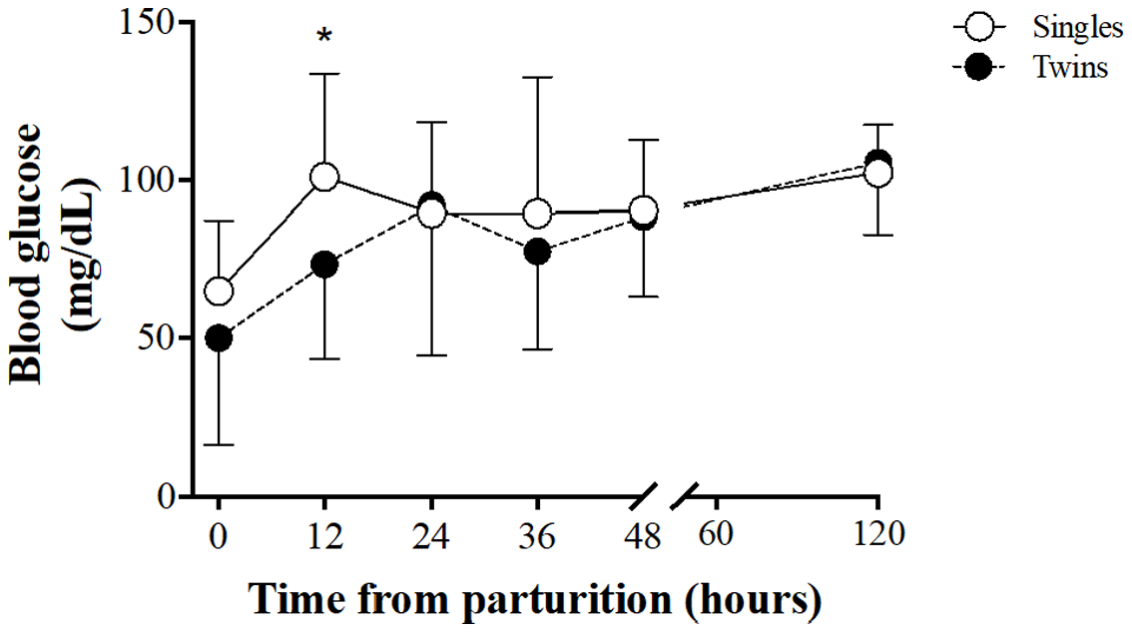
Glucose concentrations in blood of single and twin newborns during the first 5 days of life, in a model of ewe pregnancy under natural undernutrition. Asterisk indicate significant difference (*p ≤ 0.05*) in IgG in lamb’s blood plasma between groups at the same sampling time.

Assessment of blood glucose concentrations in lambs over time (Figure 3) showed similar patterns between groups, but significantly higher values of glycaemia in single than in twin lambs at 12 h postpartum (102.2 ± 32.8 vs. 73.4 ± 29.9 mg/dL, *p < 0.05*).

Cortisol concentrations in newborn blood plasma were only measured at birth and the values showed a high variability and a trend to be higher in singletons than in twins lambs (97.5 ± 15.7 vs. 72.6 ± 22.1 ng/mL, respectively; *p = 0.09*).

### 3.5. IgG concentration in colostrum and in newborns blood

**Figure 4:**
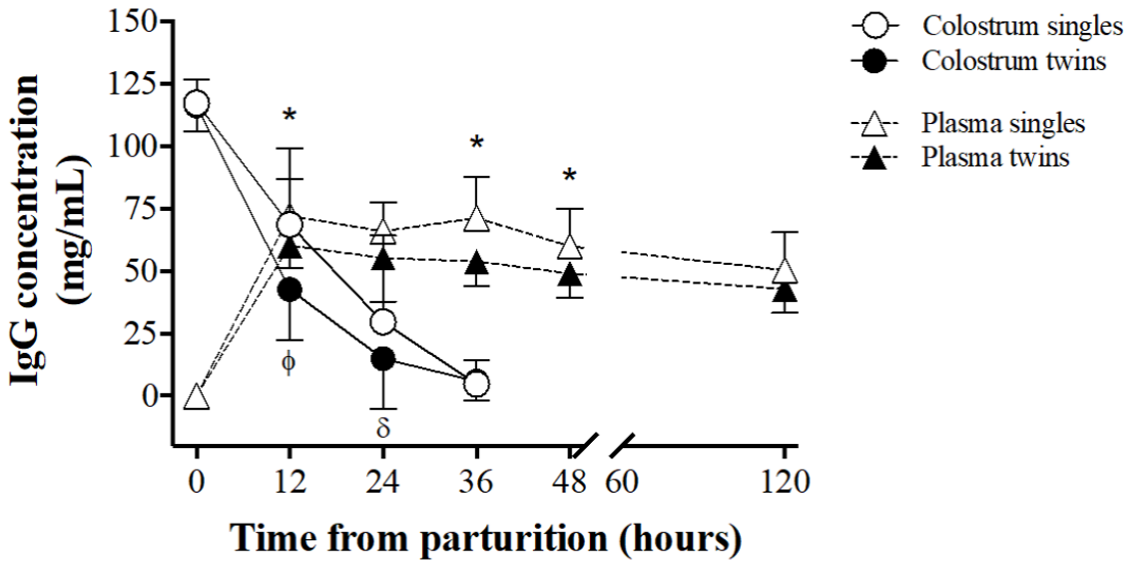
Comparison of IgG concentration patterns in maternal colostrum and in the blood plasma of newborn lambs, between singleton and twin lambings, in a model of pregnancy under natural undernutrition. Asterisks indicate significant difference (*p ≤ 0.05*) in IgG in lamb’s blood plasma between groups at the same sampling time. Greek letters indicate significant difference (*p ≤ 0.05*) in IgG content in colostrum between groups at the same sampling time.

The IgG content in both colostrum and newborns blood plasma are shown in Figure 4. Colostrum IgG content was similar at delivery in single and twin pregnancies (117.3 ± 9.5 and 116.6 ± 10.3 mg/mL, respectively). After that, IgG concentrations were significantly higher in the colostrum of single lambing ewes at 12 and 24 h (*p < 0.05*) and decreased afterwards in both groups for reaching similar values at 36 h (4.7 ± 9.7 and 5.8 ± 7.7 mg/mL in single and twin pregnancies, respectively).

The IgG concentrations in blood plasma of the newborn lambs was null at birth in both groups and increased rapidly for reaching maximum concentrations at 12 h, which were significantly higher in singleton lambs (72.1 ± 14.6 and 60.3 ± 9.3 mg/mL, for singles and twins respectively, *p < 0.05*). There was afterward a slow and gradual reduction in IgG content in both groups but differences remained for the first 48 h of life, being statistically significant at 36 and 48 h of life (*p < 0.05*).

### 3.6. Newborn lamb’s BW and mortality

The BW and mortality of newborns during the first 30 days of life are shown in the Table 3. Singleton lambs had a significantly higher BW at birth (*p < 0.05*) and such difference was maintained and even increased during the first month of life since average daily weight gain was 124.3 g/day for single lambs and 62.0 g/day for the twins (*p < 0.05*). Consequently, BW was 21.6, 32.2, and 34.5% higher in singleton than in twins at birth, 7 and 30 days of life, respectively.

**Table 3:** Body weight and mortality of single and twin newborns during the first months of life, in a model of intrauterine growth under natural maternal undernutrition.

	Body weight (Kg)			Mortality (%)		
	At birth	7 days	30 days	1–2 days	7 days	30 days
Singles	4.49 ± 0.43	5.58 ± 0.50	8.22 ± 1.83	7.1	14.3	14.3
Twins	3.52 ± 0.46	3.78 ± 0.68	5.38 ± 1.19	5.0	20.0	45.0
*p value*	*< 0.001*	*< 0.001*	*< 0.001*	*ns*	*ns*	*0.05*

ns, not significant.

Mortality of lambs, in spite of being numerically higher in singletons during the first 7 days of life, was finally 3-fold higher in twins (*p = 0.05*).

## 4. Discussion

The present study, to the best of our knowledge, is the first trial comparing production and composition of colostrum and glucose and IgG concentration in the lambs of ewes carrying single and twin pregnancies at a harsh environment, Chilean southern Patagonia, as a model for other regions worldwide.

### 4.1. Ewes BW and BCS

Assessment of BW and BCS in the sheep of our trial confirms the state of undernutrition of the sheep in the Chilean Patagonia. According to Russell’s seminal study (19), a single-bearing ewe under good nutritional conditions should increase around 10% of its BW in the last 4 weeks of gestation, while one carrying twins should increase its BW by around 18%. Likewise, the BCS should not decrease more than 0.5 points in the same period, regardless of the number of fetuses carrying by the ewe. In contrast, in our study, the ewes carrying a singleton decreased their BW along pregnancy, for reaching prior to delivery a BW 9.6% lower than at beginning of pregnancy. Twin-bearing ewes, maintained their BW, with 1% lower BW at the end of pregnancy. Our ewes started the pregnancy with a very low BCS (around 2.1) and, both in single- and twin-bearing ewes, a drastic BCS drop was observed from day 60 of gestation onwards for reaching the end of gestation with a decrease of 0.5 and 0.6 points in single- and twin-bearing ewes, respectively. These changes in BW and BCS during gestation evidence the occurrence, under these conditions, of natural maternal undernourishment, resulting in an intrauterine growth restriction and low birth-weight of the newborns (20).

### 4.2. Maternal glycemia and reproductive hormones

In well-nourished pregnant ewes, either carrying singletons or twin fetuses, blood glucose concentration range is around 29–59 mg/dL during gestation (21), and around 40–60 mg/dL near term (22). The values recorded in our study for maternal glycaemia 1 week before delivery were in the range to those previously described in animals under a severe nutritional restriction [sheep with only 25% of the daily ration for 24–48 h (22)] in which glycaemia decrease to around 41 and 28 mg/dL for single- and twin-bearing ewes, respectively. Blood glucose content in our study increased in the peripartum to more than 3-fold higher than 1 week before parturition, again without differences due to the type of parturition. This significant increase in postpartum glycaemia, albeit to a lesser extent, has also been observed in well-nourished ewes, without differences between type of parturition, and it has been associated with the stress of parturition. In fact, there is a positive correlation between maternal blood glucose and cortisol concentrations (18, 23), which is consistent with such hypothesis.

Plasma progesterone and 17β-estradiol concentrations at 140 days of gestation were higher than those reported in the literature for ewes in good nutritional status at the same gestational age (24). In addition, the twin-bearing ewes had 22% more progesterone and 13% more 17β-estradiol compared to the single-bearing ewes, which represented a statistical trend (*p = 0.061*) only for progesterone. These differences between single- and twin-bearing are lesser to that reported in well-nourished sheep (24). After lambing, progesterone and 17β-estradiol concentrations in single lambing ewes decline rapidly during the first 2 h, when progesterone reaches its basal levels. Estradiol-17β, on the other hand, continues to decrease until 24 h post lambing (25). In our trial, the concentrations of both hormones measured within the first hour postpartum were also higher than expected. A possible explanation may be related with previous reports addressing that undernutrition causes increases in blood concentrations of reproductive steroids in pregnant ewes, due to alterations in their liver clearance (26, 27).

### 4.3. Colostrum yield and nutritional composition

The amount of colostrum produced during the first 24 h after lambing is widely recognized as essential for lamb’s survival (18, 28) and, under normal nutritional conditions, colostrum yield is higher in twin-lambing ewes than in single-lambing (28) and even higher when ewes are supplemented with easily digestible carbohydrate sources at the end of pregnancy (10, 18). Conversely, the cumulative colostrum yield in our ewes during the period of study was 15.4% higher in single-lambing than in twin-lambing ewes and even higher (20%) when taking into account only the first 24 h after lambing, which reinforces evidences of chronic maternal undernutrition and clearly shows the effects on colostrum yields. Considering a basal requirement of 50 mL of colostrum per kg of live weight of lamb at birth (29), or 180–290 mL per kg of live weight during the first 18 h of life (30), it is evident that in twins this is not achieved in the current study, which seriously limits their survival probability.

We have not found information on colostrum production in ewes under chronic undernourishment. However, nutritional restriction (60% of requirements) from 50 days of pregnancy onward in single bearing ewes reduced 43% the colostrum yield measured 3 h after lambing (11), with colostrum volume comparable to those estimated for the same period in our study. A similar result was also observed in ewe-lambs with 50% restriction from 40 days of gestation (31). Furthermore, a 50% decreased colostrum yield during the first 18 h after lambing was observed in twin-bearing Scottish Blackface ewes, subjected to severe nutritional restriction during the last month of pregnancy (12). In these animals the average colostrum production at 18 h was 994 mL, while in our study the average of colostrum produced at 24 h was 25% lower. Overall, these considerations support that chronic undernutrition affects colostrum production to a greater extent in twin-bearing ewes than single-bearing ones, independently of the length of nutritional restriction period. Therefore, the effects of number of fetuses and nutritional restriction on mammary gland development and function (11, 28, 32) may explain the low volumes of colostrum found in our trial.

The nutritional components of sheep colostrum show a broad variability between breeds, with a trend to higher fat and protein concentrations in meat-type breeds. However, regardless of breed, high fat and low lactose content has been described in twin-lambing ewes (33). On the contrary, our results showed no differences in fat content and a decrease in protein and lactose content, with values about 3-fold and 2-fold lesser, respectively, than reported mean values. Studies carried out in the same breed than in the present study (Corriedale) but in good nutritional state reported no differences between twin- and single-lambing ewes in fat, protein, and lactose content of the colostrum; however, in both types of pregnancies, fat and protein values were about two-folds higher, while lactose was about half, than in our study (10, 18).

Maternal nutritional restriction (60% of requirements) from day 50 of gestation in single-bearing ewes has shown no effects in any of the nutritional components of colostrum at 3 h postpartum (11). The same findings were reported in twin pregnancies restricted from day 105 of gestation (12). The assessment of colostrum components in our study showed lower amounts of fat and protein and higher lactose than in the trial of Swanson et al. (11). These results reinforce again the evidences of a profound maternal metabolic imbalance as a consequence of chronic undernourishment. The high concentration of lactose in the colostrum of our ewes is perhaps the most notable change regarding to those reported in the literature. It is known that in animals with nutritional restriction insulin concentrations drop (34), which adds to the reduction that precedes lambing and, as a consequence, the use and storage of glucose in various tissues such as liver and muscle decreases, leaving a greater amount of glucose available for lactose synthesis in the mammary gland (35). Hence, there is a strong effort of underfed sheep for nourishing their lambs.

However, we can infer that, despite the high lactose content, the lack of fat in colostrum makes it deficient as an energy source, hindering the thermoregulation of lambs, thus conditioning their survival. In fact, taking together the nutrients present in the colostrum at lambing, the estimation of its energy value (36) was ~7.0 MJ/Kg of colostrum in single-lambing ewes and 6.4 MJ/Kg of colostrum in the twin-lambing ones, both values lower than the 8.8 MJ/Kg of colostrum reported for well-nourished single-lambing ewes grazing on improved pastures during gestation (37).

### 4.4. IgG concentration in colostrum and in newborns blood

The colostrum IgG concentration observed in our model of maternal chronic undernourishment decreases gradual but rapidly within the first 36 h postpartum, independently of type of pregnancy. However, although the IgG concentration in the colostrum was similar immediately after lambing, as previously reported (38), the drop in IgG after delivery was faster in the twin-lambing ewes; especially between 12 and 24 h postpartum. Hence, the average IgG concentration during the first 36 h postpartum was around 25% lower in the twin-lambing than in single-lambing sheep. On the contrary, reports in well-nourished ewes indicate that IgG concentration at lambing is higher in the colostrum of twin-bearing ewes during the first 24 h postpartum (39). Nutritional restriction during the last month of gestation in twin-bearing ewes had no effect on colostrum IgG concentration within 18 h postpartum, but significantly decreased total IgG production, due to lower colostrum production (40).

A large study in twin ewes of various European meat sheep breeds showed that IgG production within the first hour postpartum was between 33 and 48 g, decreasing to 10–17 g at 18 h after lambing (41). This same trend was observed in single lambing Karakul ewes, although with slightly higher initial values (59 g) (42). We found in our study an IgG production of 26 g in twin-lambing ewes and 30 g in single-lambing ones at lambing, which decreased to 11 and 5 g at 24 h postpartum for twin and singleton pregnancies, respectively.

The concentration of IgG in the plasma of newborns drastically increased in the first 12 h of life, remaining virtually unchanged until our last sampling at 120 h after birth. Plasma IgG concentration was always lower in twin lambs, potentially driven by the lower amount of colostrum and IgG of the sheep. Another plausible explanation could be that, the greater restriction suffered by the twins during gestation, may have altered the normal development and maturation of the gastrointestinal system, affecting in turns the absorption of immunoglobulins (43). In our study, plasma IgG concentration at 24 h of life in single lambs was 16% higher than in twins, in agreement with previous studies in European meat breeds (44); such difference was almost doubled in lambs of Santa Ines breed (38).

### 4.5. Newborns blood cortisol and glucose

In our study, plasma cortisol concentration at birth of singletons lambs was 25% higher than in twins. Values for both singleton and twins were in the range (45, 46) or in slightly lower values than those previously reported (47) but, in previous studies in well-nourished pregnancies, similar blood cortisol concentrations has been reported for singletons and twins (48). These differences in cortisol values between singletons and twin newborns from undernourished pregnancies in our study may be explained by different maturation of the HPA axis during the fetal life, which results in decreased adrenal sensitivity to ACTH in the late-gestation twin (49). On the other hand, maternal nutritional restriction at early gestation in single-bearing ewes showed higher plasma cortisol concentration in its litter than those from well-nourished ewes (46), although others have not found differences due to the maternal nutritional plane (50). In our study, it was evident that maternal nutritional restriction had greater effects on the growth of the twins, so their lower plasma cortisol concentration could be due not only to litter size, but also to their decreased body growth. Consistently with the above, an inverse relationship between plasma cortisol and birth weight has been previously described (47).

In the case of plasma glucose concentration, a steeper increase was observed after birth, peaking earlier in single lambs than in twins (12 vs. 24 h after birth, respectively), for stabilizing later without differences between groups after 24 h. Moreover, the blood glucose concentrations at 12 h after birth were significantly higher in the singletons. A higher glycaemia after birth has been described in single lambs than in twins of well-nourished sheep (48, 51) and, coincidentally with our results, there is a significant increase in glycaemia of single lambs from well-nourished mothers, starting around 4 h after birth and reaching its maximum at 12 h (52). The lowest glycaemia observed at the beginning of lactation in the twins may be related to placental insufficiency at the end of pregnancy (53) and lower total availability of colostrum with lower content of nutrients. This scenario may be worsened by a lower hepatic or muscular gluconeogenic capacity in low birth-weight lambs, as previously described (54).

### 4.6. Newborn lamb’s BW and mortality

Our data support previous evidences of higher birth-weight of singleton lambs in both underfed (20) and well-fed pregnancies (55–57), compared to twin-born lambs. A low birth-weight of twin lambs is the result of intrauterine growth restriction associated with a reduced placental development (i.e., reduced placentome number and total placental weight per fetus, as well as decreased total placental vascularity) leading to reduced placental nutrient transport (57). After birth, body weight gain was also affected in twin lambs, increasing the differences with singletons. These differences have also been observed in twin lambs born under adequate maternal nutrition, remaining until weaning at 99 days (58, 59). We were not able to evaluate the lamb’s weight at weaning in the present study but, in a previous study form our group, the weaning weight of twin lambs at 120 days of life was severely affected by restrictive maternal nutrition (6).

In our model of pregnancy under natural undernourishment, the type of lambing did not show a significant effect on neonatal mortality in the first 2 days of life. In single lambs, mortality only increased during the first week, while in twins there was a significant increase up to 30 days of life, being 3-fold higher than that of single lambs reaching 45%. Neonatal mortality is estimated between 15 and 20% in different herds around the world, but it is highly variable between herds; where the main causes are associated to litter size, birth weight, starvation/mismothering/infections, and cold exposure (8, 60). The difference in mortality between single and twin lambs in our study is consistent with that reported in a large study in Australia, where the mortality of twins is about 2–3 times higher than that of singles (60). In a study previously carried out in Magellan Patagonia, we observed a mortality of 29% in twin lambs at 60 days of life (6), which confirm the high variability of this trait despite of similar ewe management and breed conditions.

## 5. Conclusion

Undernourishment in pregnant ewes affected colostrum quantity and quality, which compromises the supply of colostrum and its protein, lactose and IgG for each newborn lamb in the case of twin gestations, and thereby its development and postnatal survival. Given the importance of prolificacy as a mean to increase the productivity of sheep herds, it is of great interest to search for management alternatives favoring fetal growth, birth weight and neonatal viability in twin sheep pregnancies, mainly when flocks are breed under grazing on restrictive prairies in harsh environments.

## Data availability statement

The raw data supporting the conclusions of this article will be made available by the authors, without undue reservation.

## Ethics statement

The animal study was approved by Bioethics Committee of the Faculty of Veterinary, University of Chile, and the Institutional Committee for the Care and Use of Animals (Certification # CICUA 20394-VET-UCH). The study was conducted in accordance with the local legislation and institutional requirements.

## Author contributions

JT: Conceptualization, Formal Analysis, Investigation, Writing – review & editing. FS: Conceptualization, Funding acquisition, Investigation, Writing – review & editing. OP: Investigation, Writing – review & editing. MR: Investigation, Writing – review & editing. CB: Investigation, Writing – review & editing. AC: Investigation, Writing – review & editing. AG-B: Writing – original draft, Writing – review & editing. VP: Conceptualization, Formal Analysis, Funding acquisition, Investigation, Methodology, Project administration, Supervision, Writing – original draft, Writing – review & editing.

## Funding

The author(s) declare financial support was received for the research, authorship, and/or publication of this article. This research was partially funded by Project FONDECYT 1160892, from National Agency for Research and Development (ANID), Chile; and Project ENL-2019, Research and Development Vice-Rectory (VID), University of Chile and Agricultural Research Institute (INIA-Kampenaike), Ministry of Agriculture, Government of Chile. The publication fee of this article was partially financed by the Research Department of the Faculty of Veterinary Sciences (DI-FAVET), University of Chile.

## Conflict of interest

The authors declare that the research was conducted in the absence of any commercial or financial relationships that could be construed as a potential conflict of interest.

## Publisher’s note

All claims expressed in this article are solely those of the authors and do not necessarily represent those of their affiliated organizations, or those of the publisher, the editors and the reviewers. Any product that may be evaluated in this article, or claim that may be made by its manufacturer, is not guaranteed or endorsed by the publisher.
